# The effect of knowledge, attitude, and practice model-based health education on psychological well-being and self-efficacy of patients with concurrent cerebrovascular stenosis and coronary heart disease: a quasi-experimental study

**DOI:** 10.3389/fpubh.2024.1484210

**Published:** 2025-01-30

**Authors:** Qiyu Zhou, Yuli Qian, Dan Zhang, Huan Xu, Bei Yuan, Wenfeng Tian, Qiong Li

**Affiliations:** ^1^Cardiovascular Medicine, Hubei No.3 People’s Hospital of Jianghan University, Wuhan, China; ^2^Department of Neurology, The Third People’s Hospital of Hubei Province, Wuhan, China; ^3^Third People’s Hospital Affiliated to University of Cardiovascular Medicine, Wuhan, China

**Keywords:** health education, cerebrovascular stenosis complicated with coronary heart disease, interventional therapy, knowledge, attitude, practice

## Abstract

**Objective:**

This research aimed to evaluate the effect of knowledge, attitude, and practice model-based health education on patients with cerebrovascular stenosis and coronary heart disease who underwent simultaneous interventional therapy.

**Methods:**

Sixty patients with cerebrovascular stenosis complicated with coronary heart disease and treated in The Third People’s Hospital of Hubei Province, from February 2019 to April 2021 participated in the study. Patients were randomly assigned to either a control group (*n* = 30) or an intervention group (*n* = 30). The control group received routine nursing care, while the intervention group received knowledge, attitude, and practice-based health education. Comparisons were made between the two groups regarding satisfaction rates, anxiety, depression, knowledge, attitude, practice scores.

**Results:**

The intervention group exhibited a higher satisfaction level than the control group. Additionally, the intervention group showed improved self-efficacy and reduced anxiety and depression scores at discharge, 1 month, and 3 months after discharge. The intervention group achieved higher knowledge, attitude, and practice scores at all three time points compared to the control group (*p* < 0.05).

**Conclusion:**

The results suggest that the application of Knowledge, Attitude, and Practice Model in patients with cerebrovascular stenosis and coronary heart disease can effectively enhance psychological well-being, improve self-efficacy, and enhance understanding of the disease.

## Introduction

Cardiovascular diseases are the leading cause of death worldwide (1), claiming over 17 million lives annually. Coronary heart disease (CHD) is the most common type, affecting 6.8% of U.S. adults (2). While advancements in treatment and prevention have reduced CHD mortality, many individuals require post-cardiac event support and education to manage lifestyle-related risk factors (2, 3). Despite its significant impact, awareness about cardiovascular diseases remains low. Key risk factors include physical inactivity, tobacco and alcohol use, and unhealthy diets (4, 5).

Patients with cardiovascular diseases often face significant mental health challenges. These conditions can be life-threatening, and patients must quickly adapt their lifestyle to improve their health (5). Self-care, which involves conscious actions to maintain and improve health, is crucial for these patients. The American Heart Association recommends lifestyle changes such as smoking cessation, regular check-ups, medication adherence, healthy eating, physical activity, and weight management to prevent future cardiac events. Unfortunately, adherence to these self-care behaviors often declines over time (6).

Patients often begin self-care behaviors after surgery but gradually decrease adherence within 6 months. While self-care is essential, patients often prioritize it inadequately. Patient education empowers individuals to actively participate in their self-care and manage their condition effectively. The World Health Organization recognizes patient education as an important strategy to improve patient engagement in disease management. Effective educational programs use behavior change theories and appropriate educational techniques to address factors like knowledge, beliefs, interpersonal influences, which significantly impact self-care outcomes in patients with cardiovascular diseases (6).

Patient education involves healthcare professionals providing information to patients to improve their health behaviors or overall health (7). Informed patients can better monitor their symptoms and condition. Nursing interventions can improve disease awareness, health literacy, and promote healthy behaviors (8).

Coronary heart disease can lead to psychological illness due to the burden of managing the condition. The adoption of healthy lifestyles is crucial, but patients may experience depression, anxiety, or stress when facing challenges. Unfortunately, there are minimal interventions to address these psychological concerns. Additionally, patients often lack social support, which is essential for managing the condition. Healthcare providers may also have limited training in providing quality mental health care for patients with cardiovascular diseases (5).

Lifestyle modification is crucial for managing cardiovascular disease. Factors such as knowledge, attitudes, practices (KAP), social support, and individual characteristics influence the adoption of healthy behaviors. The KAP model is a useful framework for implementing behavioral changes. By understanding the cognitive and emotional factors that influence behavior, healthcare providers can effectively support patients in adopting healthy lifestyles (9).

The Knowledge, Attitude, and Practice (KAP) model is a rehabilitation guidance model that helps analyze patients’ thoughts and behaviors. By understanding their cognition, attitudes, and actions, healthcare professionals can guide patients to identify and modify rigid behaviors and explore underlying beliefs. This model can help restore patients’ complete thinking logic. Therefore, this study aimed to explore the effect of combining the KAP model with motivational interviewing to identify and improve health education in female patients with coronary heart disease (8).

A study by Kang et al. (8) found that combining the KAP model with motivational interviewing is effective in managing systemic lupus erythematosus (SLE). This research improved patient adherence to treatment and disease management capabilities (8). Similarly, a study by Xiong et al. (9) demonstrated the effectiveness of the KAP model in managing complex conditions such as acute pancreatitis and metabolic syndrome. This model promotes healthy lifestyles, reduces complications, and improves treatment adherence, making it applicable to various conditions (9).

Cardiovascular diseases are major global health concerns, requiring a comprehensive approach to address both physical and psychological aspects of care. Concurrent conditions such as cerebrovascular stenosis and coronary artery disease pose significant challenges for patients. Knowledge, Attitude, and Practice (KAP)-based health education models offer a promising approach for improving the psychological well-being of patients. By enhancing their understanding and self-efficacy, these interventions can reduce complications, improve satisfaction, and empower patients to manage their conditions effectively.

## Methods

### Study design, setting, and participants

This quasi-experimental study was conducted on patients with cerebrovascular stenosis complicated with CHD in Hubei Provincial Third People’s Hospital affiliated to Jianghan University of Cardiovascular Medicine, China. The inclusion criteria for this study were as follows: (1) Age between 18 and 80 years old, (2) Confirmed cerebrovascular stenosis with CHD through total cerebral angiography and coronary angiography, with asymptomatic cerebrovascular stenosis ≥70% or symptomatic cerebrovascular stenosis ≥50%, and coronary artery stenosis ≥70%. CHD symptoms such as angina pectoris were not taken into consideration, but TIMI was classified as grade 1 or grade 2, (3) Angiography indicated that the cerebral and cardiovascular stenosis could be treated with interventional therapy, and after comprehensive discussion prior to the procedure, it was determined that combined interventional therapy was suitable, (4) Left ventricular ejection fraction (LVEF) was ≥30%. The exclusion criteria for this study were as follows: (1) Presence of other serious craniocerebral and cardiac diseases, (2) Surgical contraindications due to diseases of other organs and systems, (3) Severe bleeding or recent hemorrhagic diseases, (4) Contraindications to other related interventional therapies. Power analysis calculations with G*Power software indicate that (power = 80%, *p* = 0.05, number of groups = 2, and number of measurements = 4) 54 participants would be needed to detect an effect size of 0.4. Totally, 60 eligible participants allocated to the two groups (30 in each group).

### Data collection and intervention

**Figure 1:**
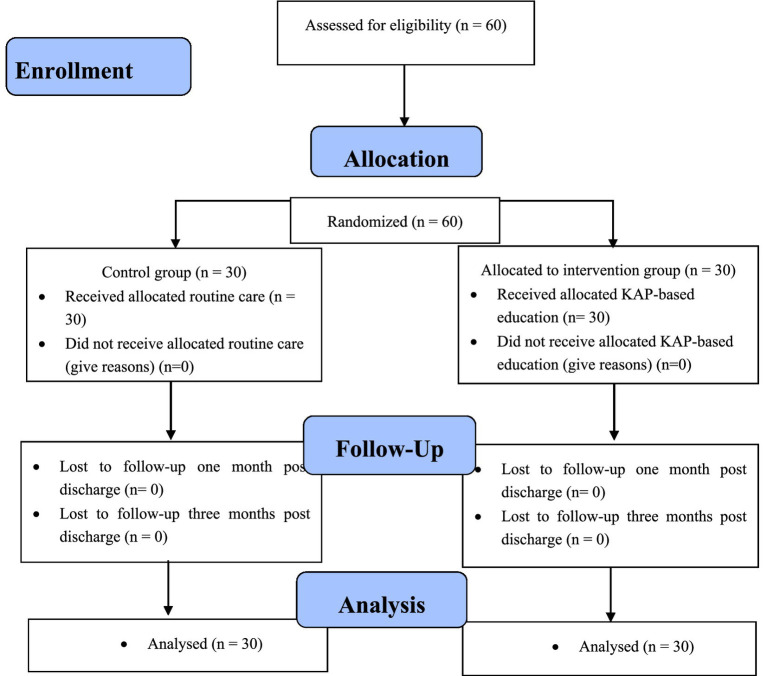
Flow chart of the study.

Participants were randomly assigned to either the intervention group (*n* = 30) or the control group (*n* = 30) using a computer-generated random number table. Randomization was conducted by an independent researcher not involved in the intervention or data collection to minimize allocation bias and ensure the integrity of the process (Figure 1).

#### Control group

Upon admission, all patients received routine treatment consisting of oral medications, dual antiplatelet therapy, and statins. This included daily administration of aspirin enteric-coated tablets (100 mg), clopidogrel sulfate tablets (75 mg), and rosuvastatin calcium tablets (10 mg) taken at night, for a minimum duration of 5 days (10). Laboratory tests were conducted to assess the patients’ condition, and symptomatic treatment was provided as needed. High-risk factors were controlled, blood pressure was maintained at approximately 120–140/60-90 mmHg, and a clear diagnosis for simultaneous treatment was established. Neurosurgeons and cardiologists collaborated to develop an individualized operation plan. A detailed treatment plan was developed, considering factors such as preparation for the procedure, treatment site, risk assessment, and perioperative care. An experienced neurosurgeon and a cardiologist carried out the simultaneous treatment. The femoral artery sheath was inserted using the Seldinger method, and the procedures were typically performed in a specific order. Brain protective devices were used during carotid artery stenting (CAS) to prevent complications. Systemic heparinization and Tirofiban were administered to prevent strokes., with an initial intravenous injection of 10–25 μg/kg, followed by a drip of 0.15 μg/(kg·min) (11). Post-operation, fluid replacement consisting of 1,000 mL of crystal solution and 1,000 mL of colloid solution was administered to facilitate the metabolism of contrast medium. Low molecular weight heparin was administered as an anticoagulant for 3 days. Dual antihypertensive therapy and statins continued until 3 months after the operation. Blood pressure was strictly controlled within a specific range using antihypertensive e drugs as needed. Follow-up examinations were conducted.

Upon emergency admission to the hospital for percutaneous coronary intervention (PCI), patients in the control group received routine cardiology care. They provided informed consent and completed questionnaires. The team members provided guidance on various aspects, including:

- Disease education: Providing information about acute myocardial infarction (AMI), medication management, smoking cessation, and the benefits of cardiac rehabilitation.
- Post-operative education: Conducting a second health education session to guide patients in adopting healthier lifestyles, managing high-risk factors, and participating in rehabilitation exercises.
- Discharge guidance: Providing information about follow-up visits, post-discharge rehabilitation, and a follow-up manual.
- Post-discharge follow-up: Regularly monitoring patients’ progress and providing guidance.

#### Intervention group

Based on the knowledge, belief, and practice model, the intervention group implemented a health education program. Specific measures undertaken in this situation included:

#### In-hospital intervention program

Explaining the study objectives, methods, and content to the patient and their family, obtaining informed consent and cooperation, administrating a questionnaire to the patient, ensuring the patient’s stability and absence of any deteriorating conditions before participation. See the health education and training program in Table 1.

**Table 1:** Summary of the intervention program based on the KAP model.

Phases	Program	Sessions/time	Content
In-hospital intervention program	Education	First	Covered the mechanism and causes of AMI, heart function, post-PCI activity plans, medication knowledge, and the benefits of cardiac rehabilitation. Health education manual: Provided detailed information on heart anatomy, common heart diseases, cardiac rehabilitation prescriptions, and cardiopulmonary resuscitation. WeChat group: Provided ongoing health education and distributed a follow-up manual for acute coronary syndrome.
		Second	Focused on promoting lifestyle changes, including reducing sedentary behavior, quitting smoking, adopting healthy diet, and maintaining a positive mindset. WeChat group: Provided ongoing health education and distributed a follow-up manual for acute coronary syndrome.
		Third	Covered post-discharge considerations such as medication adherence, follow-up appointments, self-monitoring of blood pressure and heart rate at home, and home rehabilitation. WeChat group: Provided ongoing health education and distributed a follow-up manual for acute coronary syndrome.
	Practice	During hospitalization	Patients were encouraged to discuss healthy lifestyle and identify their personal motivations based on their habits. Patients were guided on rehabilitation exercises and self-monitoring of heart rate. Inactive patients were encouraged to incorporate 30 min of daily exercise Smoking cessation guidance was provided for smokers, including resources and support. Non-smokers were advised to avoid secondhand smoke. Patients experiencing anxiety or depression were encouraged to practice self-regulation techniques such as listening to music or reading, to promote a balanced state of mind and healthy living habits. These discussions aimed to shift patients’ perspectives and promote a belief in a healthy life. By instilling this belief, patients were empowered to adopt healthy behaviors post-recovery.
Out-of-hospital intervention program	Education	Weekly (up to 3 months post discharge)	Shared weekly health information through videos, texts, and slides, covered topics like: Cardiac rehabilitation prescriptions, Healthy eating, Exercise, Heart stents, blood lipid and sugar management, Post-MI mood and sleep, Medications, Smoking cessation, and Workplace return.
	Practice	Weekly (up to 3 months post discharge)	Personalized diet plan based on individual dietary habits and needs, emphasis on sugar and fat control for hypertensive patients, encouragement of blood sugar and lipid management, dietary changes, and self-management skills, choice of exercise programs based on individual preferences and circumstances. Daily monitoring of work and exercise, promotion of at least 4–5 weekly exercise sessions. WeChat group for mutual supervision and support, gradual return to work based on the job nature and intensity. Evaluation of self-management of hypertension, smoking, and hyperglycemia, recognition and encouragement for effective management of risk factors, and identification and intervention for poor management.

#### Out-of-hospital intervention program

Health knowledge education and training plane continued 3 months post discharged. Patient also were encouraged to ask their questions and answered inquiries on Wednesdays and Fridays, provided further assistance during outpatient appointments, administered questionnaires at one and 3 months to assess progress, and provided targeted health education based on questionnaire results. In addition, to promote health belief and encourage healthy behavior, the following strategies were implemented:

Regularly health education: Shared health education knowledge in the WeChat group, customizing content based on patients’ education level, and understanding.
Informative videos and clinical cases: Enhanced understanding and provided practical insights.
Peer support: Invited patients who successfully quitted smoking to share their experiences and inspire others.
Psychological concerns: Focused on addressing anxiety, depression, and other emotions among out-of-hospital patients.
Building confidence: Promoted confidence in improving unhealthy lifestyles and prepared patients for maintaining healthy living behaviors (Table 1).

### Measures

A comprehensive literature review and expert discussions led to the development of a 10-item questionnaire to assess patient satisfaction. These items were categorized into four dimensions: very pleased, pleased, general, and displeased. The overall satisfaction rate was calculated by adding the rates of very satisfied, satisfied, and normal responses.

The Chinese Childbirth Self-Efficacy Inventory (CBSEI-C32) is a reliable and valid tool for measuring childbirth self-efficacy. It consists of two subscales: outcome expectancy (OE-16) and self-efficacy expectancy (EE-16). Each subscale is rated on a Likert scale ranging from 1 to 10. In the OE-16 subscale, 1 indicates complete unhelpfulness, while 10 represents high helpfulness. In the EE-16 subscale, 1 signifies complete uncertainty, while 10 signifies strong positivity. The total score of the CBSEI-C32 ranges from 32 to 320, with higher scores indicating higher levels of self-efficacy. The CBSEI-C32 demonstrates strong internal consistency with a Cronbach’s *α* coefficient of 0.96. It also exhibits good test–retest reliability (0.85) and construct validity with a coefficient of 0.85. Additionally, there is a strong correlation coefficient of 0.85 between the EE-16 and OEMAE-16 subscales (12, 13).

The Self-Rating Anxiety Scale (SAS) is a 20-item scale used to assess anxiety levels. The scale is divided into four grades based on the frequency of symptoms experienced by individuals. A rating of 1 indicates no or little time, 2 indicates a small part of the time, 3 indicates considerable time, and 4 indicates most or all of the time. Out of the 20 items, five (5, 9, 12, 14, 15) are scored in reverse order on a scale of 4–1, while the remaining items are scored on a scale of 1–4. To obtain a rough score, the scores of each item are added, multiplied by 1.25, and then rounded to the nearest whole number. The score range is from 25 to 100. Scores below 50 indicate no anxiety, scores between 50 and 59 indicate mild anxiety, scores between 60 and 69 indicate moderate anxiety, and scores above 69 indicate severe anxiety. The SAS is a well-accepted instrument for self-reporting anxiety levels. The scale addresses four groups of anxiety symptoms: motor, autonomic, cognitive, and central nervous system. The items reflect common symptoms of anxiety—for example, “I feel tense and anxious than usual and my hands are numb or tingling.” Of the 20 items, five items were negatively worded. According to Ramirez and Lukenbill (16), the SAS had great internal consistency reliability coefficient of 0.80. A study by Lindsay and Michie (32) found strong internal consistency (*α* = 0.92) for the SAS in people with a mental handicap. A study by Pang et al. (17) reported a Cronbach’s alpha of 0.78 and a split-half reliability of 0.75 for the Chinese version of the SAS.

The Self-Rating Depression Scale (SDS) is a tool used to assess depression levels. The scale consists of 20 items categorized into four grades based on the frequency of symptoms. A rating of 1 indicates no time or very little time, 2 indicates a small part of the time, 3 indicates considerable time, and 4 indicates most or all of the time. Out of the 20 items, ten (2, 5, 6, 10, 11, 13, 14, 17–19) are scored in reverse order on a scale of 4–1, while the remaining items are scored on a scale of 1–4. To obtain a rough score, the scores of each item are added, multiplied by 1.25, and then rounded to the nearest whole number. The score range is from 20 to 80. The Self-Rating Depression Scale (SDS) has demonstrated good internal consistency reliability, with Cronbach’s alpha coefficients ranging from 0.68 to 0.87 in different studies, including a Chinese study reporting an alpha of 0.84. This suggests that the SDS is a reliable tool for measuring depression. The Knowledge, Attitude, and Practice Level Evaluation Scale was developed by Tong Shen et al. (19) to assess patients’ knowledge, belief, and behavior regarding health education measures. The KAP questionnaire used in this study consists of 45 questions divided into three main sections: knowledge (20 questions) evaluates awareness of healthy lifestyles, cardiovascular diseases, and treatment, attitude (13 questions) examines patients’ perspectives on heart health, and practice (12 questions) measures adherence to health recommendations and behavioral changes. A four-point Likert scale was used for responses. Positive responses were scored as 1, while negative responses were scored as 0. This design simplifies analysis and converts results into percentages. Additionally, the questionnaire includes questions about patients’ financial concerns and government-supported schemes such as Medisave and Medishield.

The questionnaire is available in both English and Chinese, and a pilot test was conducted to ensure clarity and consistency. The validity and reliability of the questionnaire were confirmed by Shen et al. (19). This tool identifies gender and cultural differences in knowledge, attitudes, and practices among patients with cardiovascular diseases, and its results can contribute to the design of targeted health programs (19).

### Statistical analysis

Two individuals entered the data into Excel and conducted statistical analysis using SPSS 20.0. There was no missing data. Qualitative analysis was done using constituent ratios, and chi-square test was used for classification measures. Descriptive statistics (mean ± standard deviation) were used for describing general data. After conducting a normality test, independent sample t-tests were used to compare the control and intervention groups, while paired sample t-tests were used to compare data before and after intervention. Repeated measures ANOVA was employed for repeated measured scores before and after the intervention. Changes in indexes were observed at different time points, and Mauchly’s sphericity test was used to check the assumption of sphericity. If sphericity was met, no correction was needed for the F-test results. However, if sphericity was not met, the Greenhouse–Geisser correction was applied to adjust the degrees of freedom. The significance level for the results was set at *α* = 0.05. *p*-values less than 0.05 were considered statistically significant.

## Results

A total of 60 patients with cerebrovascular stenosis complicated with CHD were included in this study from February 2019 to April 2021. The patients were randomly assigned to either a control group or a study group. In the control group, there were 18 men and 12 women, with a mean age of 65.45 ± 3.78 years. In the research group, there were 13 men and 17 women, with a mean age of 65.44 ± 3.56 years. Overall, there were no statistically significant differences in the data (*p* > 0.05).

### Comparison of nursing satisfaction

The study group had a satisfaction rate of 100.00%, with 20 cases reporting being very pleased, 8 cases reporting being pleased, and 2 cases reporting being normal. On the other hand, the control group had a satisfaction rate of 83.33%, with 14 cases reporting being very pleased, 6 cases reporting being pleased, 5 cases reporting being normal, and 5 cases reporting being displeased. The intervention group showed significantly higher levels of nursing satisfaction compared to the control group (*p* < 0.05).

### Comparison of self-efficacy scores

Before intervention, there was no significant difference in the self-efficacy scores (*p* > 0.05). However, after intervention, the study group showed a higher self-efficacy score than the control group (*p* < 0.05) (Table 2).

**Table 2:** Comparison of self-efficacy scores [±s, points].

Group	N	OE-16		EE-16	
		Before nursing	After nursing	Before nursing	After nursing
Control group	30	122.49 ± 12.34	132.92 ± 15.44	112.59 ± 14.67	116.94 ± 10.33
Research group	30	122.77 ± 12.43	141.86 ± 15.49	112.49 ± 14.67	126.49 ± 10.89
*t*		0.087	2.238	0.026	3.484
Effect size		0.023	0.578	0.007	0.899
*P*		>0.05	<0.01	>0.05	<0.01

OE, outcome expectancy, EE, self-efficacy expectancy.

### SAS score comparison

**Figure 2:**
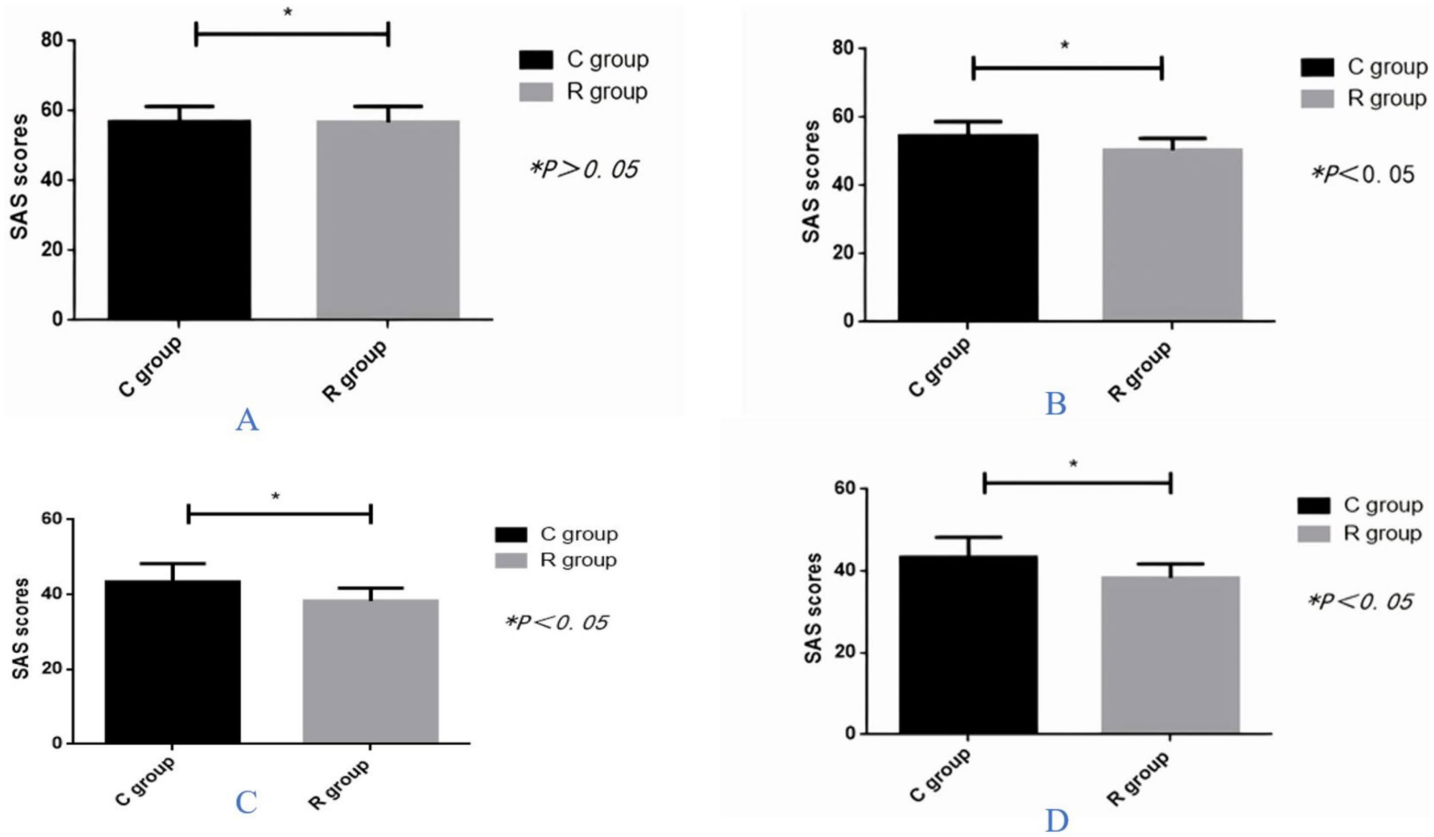
Comparison of self-rating anxiety scale (SAS) score between the two groups **(A)** before the intervention, **(B)** at time of hospital discharge, **(C)** months after hospital discharge, and **(D)** 3 months after hospital discharge.

There was no significant difference in the SAS scores before the intervention. However, the study group demonstrated a decrease in SAS scores during, 1 month, and 3 months after the intervention (Figures 2A–D).

### SDS score comparison

Before the intervention, there were no notable differences in SDS scores, but the study group showed a decrease in SDS score after the intervention, at discharge, 1 month, and 3 months after discharge. Table 3 represents all the data results.

**Table 3:** Comparison of Self-Rating Depression Scale scores [±s, points].

Group	N	Before nursing	When discharged from the hospital	One month after discharge	3 months after discharge
Control group	30	62.59 ± 3.66	59.19 ± 2.45	51.39 ± 3.34	45.29 ± 5.54
Research group	30	62.48 ± 3.74	52.93 ± 3.31	44.19 ± 3.35	38.19 ± 2.45
*t*		0.115	8.326	8.336	6.419
Effect size		0.030	2.366	2.152	1.657
*P*		>0.05	<0.01	<0.01	<0.01

### Comparison of knowledge, attitudes, and practice scores

**Figure 3:**
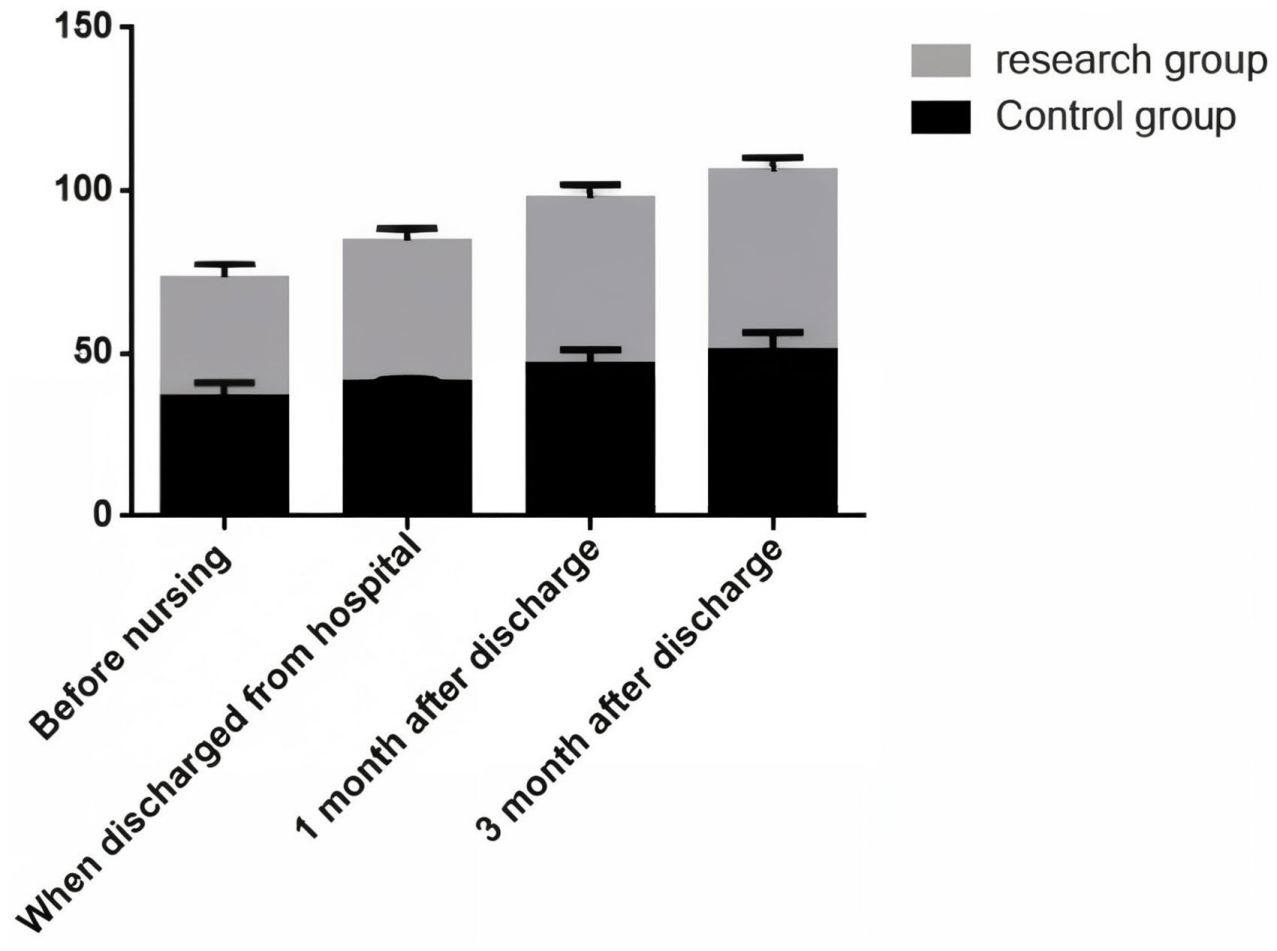
Comparison of knowledge, attitude, and practice scores between the two groups at different times.

Knowledge, attitudes, and practice scores did not show any remarkable differences before the intervention (*p* > 0.05), but the study group indicated higher scores after the intervention, at discharge, 1 month, and 3 months after discharge (*p* < 0.05) (Figure 3).

## Discussion

This study demonstrated that applying the KAP model to health education significantly improved psychological well-being of patients with cerebrovascular stenosis and coronary artery disease. Anxiety and depression levels decreased, while self-efficacy increased. These results not only confirm the impact of educational interventions on patient knowledge and awareness but also underscore the value of comprehensive and systematic methods in health education. Enhanced self-care behaviors and strengthened health beliefs were key outcomes, contributing to better treatment adherence and reduced complications.

Our study demonstrated the positive impact of interventions on patients’ self-efficacy. These improvements are consistent with research examining the impact of web-based education on the management of chronic diseases. One study indicated that increasing awareness and providing practical tools can significantly enhance self-efficacy (20). The results of this study align with a review on educational methods to improve self-efficacy in patients with coronary heart disease. The review highlighted the effectiveness of multifaceted approaches, including in-person sessions, digital tools, support groups, and informational brochures. Additionally, it emphasized the role of digital tools like mobile apps and reminders in supporting continuous adherence to treatment. These findings support the use of the KAP model alongside other personalized educational interventions, including group support, to improve patient self-efficacy, motivation, and adherence (21). Additionally, our study showed an improvement in patients’ self-care behaviors. These findings align with research highlighting the impact of education on improving treatment adherence and reducing post-surgical complications. The study of Li et al. (20) emphasized the benefits of multifaceted educational approaches for patient empowerment. Previous research has suggested that close nurse–patient interaction can improve adherence and confidence in patients undergoing CABG (22). Additionally, the KAP model encourages patients to take a more active role in disease management by consciously learning self-care skills, ultimately leading to better problem-solving compared to traditional nursing care alone (23).

The significant reduction in anxiety and depression in our intervention group is consistent with research on web-based educational interventions for patients with cardiovascular diseases. These studies show that providing targeted information and psychological support can reduce patients’ concerns (20). Patients undergoing CABG experience anxiety and fear both before and after the surgery (24). This emphasizes the importance of our study, as these anxieties can worsen patient outcomes. Our findings are consistent with those of Hoseini et al (25), who indicated the positive impact of educational interventions on reducing anxiety and depression in patients with heart disease. This study showed sustained reduction in anxiety and depression in patients undergoing coronary artery bypass graft (CABG) surgery using audiotapes as a cost-effective educational method. This reinforces the value of educational tools alongside other approaches to improve patients’ psychological well-being (25). Additionally, Aghakhani et al. (26) found that educating patients with myocardial infarction significantly reduced their anxiety and depression.

The study conducted by Kang et al. (8) also showed that combining the KAP model with motivational interviewing can effectively improve health education and chronic disease management such as lupus. This method not only increases patient adherence to treatment but also improves their ability to manage their disease (8). Most patients undergoing heart surgery experience anxiety and fear both before and after the surgery. Given the short hospital stay, it is impossible for patients to acquire all necessary knowledge and skills for disease management solely from the nursing staff (20). This can lead to a care gap after discharge, leaving patients and caregivers feeling helpless, especially in rural areas with limited access to medical facilities (27). Therefore, insufficient knowledge and lack of professional nursing guidance always result in a significant psychological burden for most patients with heart disease (28). Jin et al. (29) and Ma et al. (30) have suggested that family nursing combined with network support and specialized nursing programs can effectively improve the prognosis and reduce adverse emotions in patients undergoing CABG.

According to the KAP theory, patients who receive personalized disease-related knowledge are more likely to enhance self-care abilities, boost confidence, and ultimately translate this health-related knowledge into positive health behaviors (23, 31).

### Limitations

Despite its promising results, this study has several limitations, including a small sample size, a focus on a single geographic area, and a short follow-up period, which may limit the generalizability of the findings. Future studies should involve larger and more diverse populations, longer follow-up durations to assess long-term effects and explore individual differences such as age, gender, clinical statues, and socioeconomic status. Additionally, comparing the KAP model to other educational approaches and evaluating its economic impacts could provide more comprehensive insights for its application in healthcare systems. And also the study was conducted in a single hospital in China, limiting the generalizability of the findings to other settings or populations. Further research in diverse geographic and cultural contexts is needed.

### Implication of the study results

The findings of this study indicate that the KAP model can effectively improve patients’ self-efficacy while reducing anxiety and depression. These results can serve as a foundation for developing standardized educational programs for chronic disease management. Practical applications include designing hospital-based interventions, developing digital tools for patient education, and enhancing nurse–patient interactions. Moreover, these outcomes could contribute to reducing healthcare costs and improving the efficiency of care delivery systems.

## Conclusion

The results suggest that the application of the Knowledge, Attitude, and Practice (KAP) Model in patients with cerebrovascular stenosis and coronary heart disease is associated with improvements in psychological well-being, self-efficacy, and disease knowledge. However, due to the potential influence of biases and confounders, causality cannot be firmly established.

## Data Availability

The raw data supporting the conclusions of this article will be made available by the authors, without undue reservation.
